# Genomic Evaluation of the Genus *Coltivirus* Indicates Genetic Diversity among Colorado Tick Fever Virus Strains and Demarcation of a New Species

**DOI:** 10.3390/diseases9040092

**Published:** 2021-12-17

**Authors:** Holly R. Hughes, Jason O. Velez, Kelly Fitzpatrick, Emily H. Davis, Brandy J. Russell, Amy J. Lambert, J. Erin Staples, Aaron C. Brault

**Affiliations:** Arboviral Diseases Branch, Division of Vector-Borne Diseases, Centers for Disease Control and Prevention, Fort Collins, CO 80521, USA; jdv4@cdc.gov (J.O.V.); hwm8@cdc.gov (K.F.); qru2@cdc.gov (E.H.D.); bmk8@cdc.gov (B.J.R.); ahk7@cdc.gov (A.J.L.); auv1@cdc.gov (J.E.S.); zlu5@cdc.gov (A.C.B.)

**Keywords:** Colorado tick fever virus, *Coltivirus*, phylogeny, whole genome sequencing

## Abstract

The type species of the genus *Coltivirus*, Colorado tick fever virus (CTFV), was discovered in 1943 and is the most common tick-borne viral infection in the Western US. Despite its long history, very little is known about the molecular diversity of viruses classified within the species *Colorado tick fever coltivirus.* Previous studies have suggested genetic variants and potential serotypes of CTFV, but limited genetic sequence information is available for CTFV strains. To address this knowledge gap, we report herein the full-length genomes of five strains of CTFV, including Salmon River virus and California hare coltivirus (CTFV-Ca). The sequence from the full-length genome of Salmon River virus identified a high genetic identity to the CTFV prototype strain with >90% amino acid identity in all the segments except segment four, suggesting Salmon River virus is a strain of the species *Colorado tick fever coltivirus.* Additionally, analysis suggests that segment four has been associated with reassortment in at least one strain. The CTFV-Ca full-length genomic sequence was highly variable from the prototype CTFV in all the segments. The genome of CTFV-Ca was most similar to the Eyach virus, including similar segments six and seven. These data suggest that CTFV-Ca is not a strain of CTFV but a unique species. Additional sequence information of CTFV strains will improve the molecular surveillance tools and provide additional taxonomic resolution to this understudied virus.

## 1. Introduction

The family *Reoviridae* is comprised of two subfamilies, *Spinareovirdae* and *Sedoreoviridae* [1]. The genus *Coltivirus* is one of nine genera in the subfamily *Spinareroviridae*, with two tick-borne viruses, Colorado tick fever virus (CTFV) and Eyach virus, representing the first viruses to comprise the genus [2]. Viruses belonging in the genus *Coltivirus* have genomes made of 12 segments of double-stranded RNA, encoding for 13 viral proteins (VP) 1–12 [3]. Segment nine of coltiviruses produces two proteins, VP9 and VP9′, via a readthrough of the termination codon [4]. Colorado tick fever virus was isolated in 1943 from a febrile patient and has subsequently been found to be distributed in North America in the Rocky Mountain region between 4000 and 10,000 feet, where the vector, *Dermacentor andersoni,* is common [5,6]. Additional serotypes of CTFV have been described, including California hare coltivirus (referred to in the literature as CTFV-Ca) and Salmon River virus [7,8]. Eyach virus was isolated in Germany in 1976 from an *Ixodes* species tick [9] and was subsequently identified in France in 1981 [10]. In contrast to the febrile disease of CTFV, Eyach virus is known to cause neuroinvasive disease in humans. Recent discoveries of Kundal virus in India [11], Tarumizu tick virus in Japan [12], and Tai Forest reovirus in Cote d’Ivoire [13] have expanded the genus to include five viral species. Of these newly identified coltiviruses, Tai Forest reovirus has only been isolated from bats, and a tick vector has not been identified.

CTFV is the most commonly reported tick-borne viral disease in the Western US. Cases occur in the early spring and summer months when ticks are most active. CTFV causes a febrile illness, often with a biphasic fever and leukopenia. Up to 30% of the cases require hospitalization [14]. Life-threatening complications and deaths due to CTFV infections are rare, with fatalities associated with meningoencephalitis or disseminated intravascular coagulation. Other reported complications include meningoencephalitis, hepatitis, pericarditis, myocarditis, atypical pneumonia, disseminated intravascular coagulation, and epididymo-orchitis [14,15]. In general, the number of CTFV cases reported annually has decreased over time [14,16]. It is unclear if this reflects changes in the testing or reporting practices and certain states where the disease is found not having given it a notifiable condition. However, recent increases in case counts were seen in selected states, but it is unclear if the incidence is related to human activity or increased clinical awareness [16]. Although CTFV has been a recognized cause of human illness since the 1940s, very limited genetic information is available for CTFV, with only one complete genome sequence available for the exemplar CTFV isolate, Florio. Herein, we report the full genomes of two strains of CTFV, two strains of Salmon River virus, and one strain of CTFV-Ca. Increasing the available genetic sequences will improve our knowledge of the genetic diversity, support surveillance, and aid in optimizing molecular diagnostic testing.

## 2. Materials and Methods

All protocols followed manufacturers’ recommendations unless otherwise noted.

### 2.1. Viruses and RNA

Viruses sequenced in the study were provided by the Arboviral Diseases Branch, Division of Vector-Borne Diseases, Centers for Disease Control and Prevention’s Arbovirus Reference Collection (ARC). Isolate details are described in Table 1. RNA was extracted from frozen cell culture stocks (vero cell culture supernatant or suckling mouse brain homogenate, Table 1) using the QIAmp Viral RNA mini kit (Qiagen, Germantown, MD, USA). RNA was treated with 50% *v*/*v* dimethyl sulfoxide and incubated 1.5 h at 65 °C to denature double stranded RNA [17]. RNA was purified using an RNeasy cleanup kit (Qiagen).

### 2.2. Genome Sequencing and Assembly

The cDNA was generated from extracted RNA using random primers provided in the Ovation RNA-seq system V2 kit (Tecan, Redwood City, CA, USA). Sequencing libraries were prepared from the cDNA using the Ion Xpress plus fragment library kit (Thermo Fisher, Waltham, MD, USA), barcoded with Ion Xpress barcodes (Thermo Fisher), and quantified using the Ion library TaqMan Quantitation kit (Thermo Fisher). Sequencing templates were prepared using the Ion One Touch 2 system and Ion Hi-Q View OT2 kits (Thermo Fisher). Whole genome sequencing was performed on the Ion Torrent Personal Genomics Machine system using the Ion Hi-Q view sequencing kit and 318 v2 chips (Thermo Fisher), sequencing two libraries per chip.

Sequences (fastq) with quality phred Q > 20 were uploaded into CLC Genomic workbench v20 (Qiagen). Genomes were assembled via de novo assembly with a bubble size of the average read length (150 bp). The longest contigs with deepest coverage were submitted for BLAST analysis through NCBI. Consensus sequences for each segment were extracted from CLC genomic workbench and used for reference guided assembly in SeqMan NGen v17 (DNASTAR, Madison, WI, USA). Whole genomes were obtained for each virus with a minimum of 20× coverage, and a minimum average of 8.9 read depth per nucleotide. Putative open-reading frames were identified using EditSeq (DNASTAR). Terminal ends were determined for all sequences using 5′ RACE System 2.0 kits (Thermo Fisher) and 3′ poly(A) tailing kit (Ambion, Austin, TX, USA), followed by 3′ RACE kit (Thermo Fisher) using gene specific primers (IDT, Coralville, IA, USA). RACE DNA templates were cloned with TOPO-TA sequencing kits (Thermo Fisher) and sequenced by capillary sequencing on an ABI 3130 instrument (Thermo Fisher) using the primers provided in the TOPO-TA kits. All sequences generated in this study have been deposited into GenBank (Table 1).

### 2.3. Molecular and Phylogenetic Analysis

Conserved domains were identified in each segment using CD-search through NCBI (https://www.ncbi.nlm.nih.gov/Structure/cdd/wrpsb.cgi (accessed on 29 January 2021)). Nucleotide sequences comprising the open-reading frames for each protein were codon aligned using the Clustal W function of Mega 7 [18]. Percent sequence identity was calculated in Mega 7 using the p-distance model with G + I frequencies and complete deletion of missing data. Substitution models for each segment were determined using the model fit function of Mega 7. Bayesian inference was completed using BEAST v 1.8.4 [19] executed through the CIPRES Scientific Gateway (www.phylo.org (accessed on 1 February 2021)) [20]. A lognormal relaxed clock and a coalescent constant tree prior was used with an MCMC of 50 million generations and 10% burn-in. Priors were tested using the generalized stepping-stone sampling method [21]. Convergence of parameters was verified using TRACER v1.5 [22]. Maximum clade credibility trees were generated using TreeAnnotator [19] and FigTree V 1.4.3 showing the posterior probability of each branch. Maximum likelihood trees generated in Mega 7 were used to verify Bayesian tree topologies.

Reassortment was evaluated by concatenating the open-reading frames of 12 segments representing the complete genomes. Only reassortment events associated with a full segment of the concatenated genome that had *p* < 0.05 using the Bonferroni adjustment and detected by three or more models using Recombination Detection Program (RDP) v 4.9 [23] were considered significant [24].

### 2.4. Serological Evaluation

Positive control hyperimmune acidic fluids were diluted 1:5 in a total of 120 µL, followed by a serial 2-fold dilution with BA-1 media. Dilutions were then challenged with 100 plaque forming units of virus in a total of 60 µL, and incubated 1 hr at 37 °C. Following incubation, 100 µL of the virus with control mixture was added to a confluent Vero Cell monolayer on a 6-well plate (Corning, Corning, NY, USA) and incubated at 37 °C for 1 hr. Following incubation, 3 mL of yeast extract-lactalbumin (Ye-lah) overlay media with 0.5% (*w*/*v*) Agarose (Lonza, Basel, Switzerland) and 3% (*v*/*v*) bicarbonate (Gibco, Waltham, MD, USA) were added to each well, and plates were incubated at 37 °C. 3 mL of secondary overlay of Ye-lah media with 0.5% (*w*/*v*) agarose, 3% (*v*/*v*) bicarbonate, and 2% (*v*/*v*) neutral red (Sigma-Aldrich, St. Louis, MO, USA) was applied after an appropriate number of days to allow for differences in viral growth: 5 days for Eyach virus, 4 days for Salmon River, 4 days for CTVF-Ca, and 2 days for CTFV. Plaque counts were then noted on the following day. Titers were determined by taking the reciprocal of the highest serum dilution that reduced plaque counts by 90%, as indicated by the back titration of each virus.

## 3. Results

### 3.1. Phylogenetic Construction of the Genus Coltivirus

**Figure 1:**
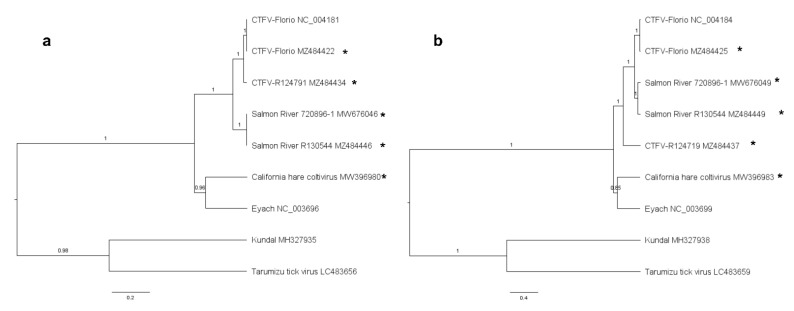
Bayesian Maximum Clade Credibility trees of Colorado tick fever virus strains and select members of the genus *Coltivirus*. Nucleotide coding sequences depicting phylogeny of (**a**) segment 1 and (**b**) segment 4. Viruses sequenced in this study are labeled with an asterisk. Taxa are labeled with virus name, strain, and GenBank accession number. Branches are labeled with the posterior probabilities, and scale bar depicts nucleotide substitutions per site. Colorado tick fever virus (CTFV).

Phylogenies were constructed using Bayesian inference. The phylogenetic tree for segment one, the putative polymerase, places the CTFV and Salmon River viruses in a well-supported monophyletic clade, while CTFV-Ca is in a clade with Eyach virus, with the Kundal and Tarimuzu Tick viruses forming a third clade (Figure 1a). Similar phylogenetic trees were inferred for all the segments (Supplementary Figure S1), except segments four and six. Trees for segments four (Figure 1b) and six (Supplementary Figure S1) place the CTFV isolate R124719 basal to the node for the CTFV clade. Recombination detection supports the placement of segment four of R124719. Taken together, these data suggest segment four of isolate R124719 is the product of a reassortment event between CTFV and an unknown donor.

### 3.2. Segment Analysis

Each segment was evaluated for size, open reading frames, and evolutionary distances. All the CTFV and Salmon River viruses sequenced in this study have consistent segment and ORF sizes (Table 2). The California hare coltivirus and Eyach viruses differ in the segment length and ORF size in segments two and seven, having different lengths compared to CTFV, Salmon River virus, and each other. The ORF of segment two is 3804-nt in length for CTFV and Salmon river virus. However, segment two of the CTFV-Ca and Eyach viruses is 3858 and 3828-nt in length, respectively. The ORF in segment seven for CTFV and Salmon River virus is 1983-nt, while the ORF of segment six for CTFV-Ca is 2097-nt, and for Eyach virus is 2100-nt in length. Conversely, the segment six ORF of CTFV and Salmon River virus is 2094-nt in length, while the ORF of segment seven in CTFV-Ca and Eyach is also 2094-nt in length. Additionally, conserved domains were identified in segment seven of CTFV-Ca, indicating the presence of nucleotide binding proteins, such as chromosome segregation proteins in amino acids 311–575. These similar regions have previously been identified in Eyach segment seven and CTFV segment six [25]. These data suggest that CTFV-Ca has a similar genetic structure to Eyach virus. Interestingly, the ORF of segment 12 was found to be 558-nt in length in CTFV, Salmon River virus, and CTFV-Ca but 555-nt in Eyach virus. Although the ORF of segment 12 in CTFV-Ca is similar in size to CTFV, phylogenetic inference placed CTFV-Ca and Eyach in a well-supported clade (Supplementary Figure S1). Of note, segment four in CTFV isolate R124719 had a longer ORF than the viruses evaluated in this study. Surprisingly, the CTFV sequenced in this study differed in size between the reference CTFV, isolate Florio, in segments two and eleven. When comparing the reference sequence (RefSeq) from CTFV–Florio–RefSeq for segment two (NC_004182) to CTFV–Florio and CTFV R124719 sequenced in this study, the reference sequence contains one nucleotide insertion at position 850, one nucleotide deletion at position 886, and one nucleotide deletion at position 3631. All three identified discrepancies were attributed to a thymine deletion or insertion in the homopolymer regions. Additionally, the CTFV–Florio–RefSeq sequence displays a 4-nt deletion beginning at position 699 for segment 11 (NC_004191) compared to the CTFV strains sequenced in this study.

Both CTFV-Ca and Salmon River viruses have the canonical terminal ends attributed to coltiviruses. The most commonly observed terminal end sequence in both viruses was 5′-GACATTTG…TTGCAGTC-3′. Although subtle differences between the segments were observed with CTFV-Ca, the overall 3′ terminal end sequences were determined to be 5′-DTGCAGTC-3′.

### 3.3. Evolutionary Distances

**Figure 2:**
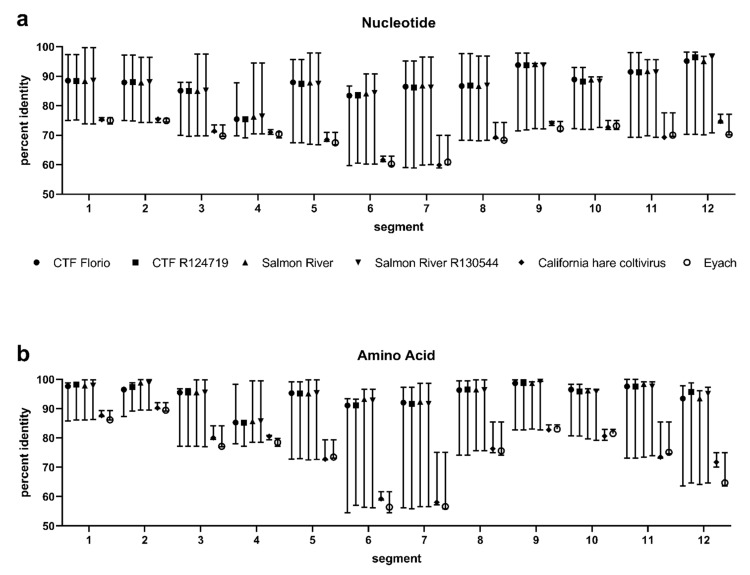
Comparison of genetic identities between Colorado tick fever virus strains and Eyach virus. Segment open-reading frames were codon aligned and percent identities were calculated for (**a**) nucleotide and (**b**) amino acids. Graphed points indicate median and range. CTF = Colorado tick fever.

Percent identities comparing nucleotides (Figure 2a) and amino acids (Figure 2b) were calculated for CTFV, Salmon River, CTFV-Ca, and Eyach viruses (Supplementary Table S1). Overall, percent identities between CTFV and Salmon River viruses are highest and ranged from 98% nucleotide identity in segment 12 to 75% in segment four. California hare coltivirus and Eyach displayed the lowest identity to CTFV and Salmon River virus, as low as 59% nucleotide identity in segment seven. Overall, amino acid percent identities were increased compared to nucleotide identities in nearly all the segments, except CTFV-Ca and Eyach exhibited lower amino acid than nucleotide identities in segments six (<59%), seven (<58%), and twelve (<71%) compared to the CTFV and Salmon River viruses. Interestingly, CTFV isolate R124719 had reduced identities in segment four compared to CTFV Florio and the Salmon River viruses. This low identity supports the segment four phylogenetic tree placing CTFV R124719 on a node basal to similar CTFV isolates and the observation of a longer ORF than other coltiviruses evaluated.

### 3.4. Serological Evaluation

Plaque reduction neutralization testing was completed for CTFV, Salmon River virus, and CTFV-Ca to understand the serological relationships (Table 3). CTFV Florio was neutralized efficiently by mouse hyperimmune acidic fluid raised against CTFV Florio (PRNT_90_ of 1:5,120) and was also cross-neutralized by Salmon River virus antiserum, although at a dilution >4-fold less than homologous neutralization (PRNT_90_ 1:320). Salmon River virus was neutralized equally by antiserum generated against CTFV Florio and Salmon River virus (PRNT_90_ 1:640). Salmon River virus was also cross-neutralized by antiserum produced against CTFV-Ca, but at a very low end-point titration (PRNT_90_ 1:20). Moreover, CTFV-Ca was only neutralized by homologous antiserum (PRNT_90_ 1:160) and CTFV-Ca antiserum did not cross-neutralize CTFV Florio or Salmon River virus. Eyach virus was cross-neutralized at very low dilutions by antiserum generated against Salmon River and CTFV Florio (PRNT_90_ 1:10) but was not neutralized by antiserum from CTFV-Ca.

## 4. Discussion

The genus *Coltivirus* has expanded in recent years to include newly described viruses as new species (Kundal virus, Tai Forest reovirus, and Tarumizu Tick virus); however, very little full-length sequence information has been available for the known viruses in the CTFV species complex, specifically CTFV, Salmon River virus, and CTFV-Ca. This study was initiated to improve the current knowledge of CTFV diversity and expand the known database of sequence information. We were able to generate full-length sequence information for two isolates of CTFV, two isolates of Salmon River virus, and the only known isolate of CTFV-Ca. Collectively, the data presented herein highlight a previously underappreciated diversity of CTFV and suggest the placement of Salmon River virus as a strain of CTFV, while CTFV-Ca likely represents a unique coltivirus species.

California hare coltivirus was isolated in California in 1976 from a hare (*Lepus californicus)* and given the designation S6-14-03 [26]. Previous studies have determined partial sequence information for this virus [25], and, here, we describe the first complete genome sequences for all 12 segments of CTFV-Ca. Phylogenetic inference places CTFV-Ca consistently in a monophyletic clade with Eyach virus for all the segments. These tree topologies are supported by evolutionary distances as CTFV-Ca is more closely related to Eyach than CTFV. California hare coltivirus also had a similar genetic structure to Eyach virus as segment seven of these viruses have similar conserved functional domains and are most related to CTFV segment six [25]. The phylogenetic inference and genetic architecture of CTFV-Ca being more similar to Eyach virus seemingly supports the theory of the evolution of European coltiviruses from North American coltivirus following the migration of host species from North America through Asia [25,27]. In our serological analysis, CTFV-Ca does not display any cross-neutralization with any coltivirus tested, which is similar to previous serological analyses [26]. Collectively, these data suggest that CTFV-Ca is not a strain of CTFV but a unique species within the genus. This conclusion is not unique and supports previous serological studies suggesting CTFV-Ca is more closely related to Eyach but is a distinct species from both CTFV and Eyach [8]. However, applying taxonomic species demarcation >55%, >57%, >60% amino acid identity in VP6, VP7, and VP12, respectively, CTFV-Ca is considered a strain of both CTFV and Eyach virus. Given this newly described genetic information, the species demarcation criteria may need to be revised for the genus *Coltivirus*.

Salmon River virus was isolated from an ill person in Idaho in 1990. Presently, Salmon River virus is an unclassified coltivirus [1]. In our analysis, Salmon River virus formed well supported monophyletic clades with reference strains of CTFV and a recent Salmon River virus isolate. This analysis is supported by the high percent identities described between the CTFV isolates and the two Salmon River virus isolates. Moreover, some cross-neutralization was observed between the two viruses, with strong neutralization of the Salmon River virus by CTFV antiserum and 4-fold less neutralization of the CTFV by Salmon River virus antiserum. While previous studies have described Salmon River virus as serologically distinct from CTFV, genetic relatedness was demonstrated [7]. The full-length sequences of Salmon River virus generated in this study confirm these previous observations and display a high degree of sequence identity between Salmon River and CTFV [7]. Using the current species demarcation criteria for the genus *Coltivirus*, Salmon River virus could be considered a strain of the species *Colorado tick fever Coltivirus.*

The highest genetic diversity between the CTFV strains was observed within segment four. The nucleotide diversity in segment four is supported by the unique phylogenetic placement of the CTFV strains compared to other segments, as well as RDP analysis, suggesting this segment was subject to reassortment. Our observation of segment reassortment is supportive of previous studies suggesting the reassortment of multiple CTFV segments in the field [28,29]. A low sequence identity in segment four was found between CTFV isolates as well as other coltiviruses, with the nucleotide percent identity averaging 77%. The low level of nucleotide identity described in this study is supported by previous studies using RNA hybridization techniques, which also determined segment four to exhibit high genetic variation between CTFV isolates [30]. Although the sequence identity to segment four was low, the amino acid identities were higher, suggesting, although the RNA displays variation, the product of the gene is under strong purifying selection pressure. The high amino acid identity is likely due to the prediction that the product of segment four is a capsid protein and responsible for essential membrane binding [31]. Overall, the percent identities across all the segments with the CTFV strains were found to be <90%, with the exception of segments nine and twelve with very high identity, suggesting that these segments may be good targets for the molecular detection of both CTFV and Salmon River viruses [7].

Of unique interest, the sequences derived in this study display sequence variation from the CTFV strains previously sequenced and deposited into public databases. The sequences derived from CTFV Florio prototype virus obtained from the Arbovirus Reference Collection differed in segments two and eleven from the published reference sequences [3]. Prior to this study, only one whole genome reference was available for CTFV. While a previous study has partially sequenced segment eleven from multiple CTFV isolates [32], the amplified region published did not overlap with the four nucleotide deletion of segment eleven data observed in this study. Additionally, the published partial sequences of CTFV-Ca isolate S6-14-03 [25] match more closely to the CTFV and Salmon River viruses sequenced in this study than to the CTFV-Ca sequences presented here. The sequences of CTFV-Ca derived here show a higher degree of relatedness to Eyach virus than to the CTFV reference sequences and CTFV sequences derived in this study, which agrees with the serological and molecular data suggesting CTFV-Ca is distinct from CTFV [7,8]. In addition, the genetic stability of coltivirus strains when subjected to passaging remains unknown and could also explain the observed diversity. Overall, these data highlight the importance of the independent evaluation of reference strain sequences and depositing virus isolates in multiple reference collections to preserve historical value [33,34]. Additionally, these data highlight the importance of the periodic sequencing of newer strains and strains from other locations to ensure the validity of molecular targets for clinical diagnostic testing.

## Figures and Tables

**Table 1:** Colorado tick fever viruses sequenced in this study obtained from the Arbovirus Reference Collection.

Virus	Isolate Designation	Date	Location	Passage	GenBank No.
Colorado tick fever	Florio	1943	Colorado	SM14	MZ484422-33
Colorado tick fever	R124719	2017	Wyoming	Vero 1	MZ484434-45
Salmon River	720896-1	1990	Idaho	SM3	MW67046-57
Salmon River	R130544	2019	Oregon	Vero 1	MZ484446-57
California hare coltivirus	S6-14-03	1976	California	SM5	MW396980-91

SM = suckling mouse brain.

**Table 2:** Segment comparison of Colorado tick fever viruses sequenced in this study and reference coltiviruses.

	Virus Nucleotide Open Reading Frame Size (Segment Size)						
Segment	CTFV-Florio-RefSeq ^1^	CTFV-Florio-ARC ^2^	CTFV R124719	Salmon River	Salmon River R130544	California Hare Coltivirus	Eyach
1	4308 (4350)	4308 (4350)	4308 (4350)	4308 (4349)	4308 (4349)	4308 (4349)	4308 (4349)
2	3630 ^4^ (3909)	3804 (3909)	3804 (3909)	3804 (3908)	3804 (3908)	3858 (3964)	3828 (3934)
3	3549 (3586)	3549 (3586)	3549 (3586)	3549 (3585)	3549 (3585)	3549 (3585)	3549 (3585)
4	3084 (3157)	3084 (3156)	3087 (3159)	3084 (3156)	3084 (3156)	3084 (3156)	3084 (3156)
5	2256 (2432)	2256 (2432)	2256 (2432)	2256 (2431)	2256 (2431)	2256 (2419)	2256 (2398)
6 ^3^	**2094 (2141)**	**2094 (2141)**	**2094 (2141)**	**2094 (2140)**	**2094 (2140)**	2097 (2175)	2100 (2178)
7	2055 (2133)	2055 (2133)	2055 (2133)	2055 (2133)	2055 (2132)	**2094 (2139)**	**2094 (2139)**
8	1983 (2029)	1983 (2029)	1983 (2029)	1983 (2028)	1983 (2028)	1983 (2029)	1983 (2028)
9	1809 (1884)	1809 (1884)	1809 (1884)	1809 (1884)	1809 (1884)	1809 (1884)	1809 (1884)
10	1818 (1880)	1818 (1880)	1818 (1880)	1818 (1880)	1818 (1880)	1818 (1879)	1818 (1879)
11	750 (998)	927 (1002)	927 (1002)	927 (1002)	927 (1002)	927 (1002)	927 (1002)
12	558 (675)	558 (675)	558 (676)	558 (675)	558 (675)	558 (678)	555 (678)

^1^ Colorado tick fever virus, Florio strain, sequences obtained from the reference sequence database (NC_004180-90). ^2^ Colorado tick fever virus, Florio strain, obtained from the Arbovirus Reference Collection (ARC). ^3^ Bold segments highlight the similarity between segments 6 of CTFV and 7 of Eyach and California hare coltivirus. ^4^ Underlined values represent differences in size between reference strains and viruses sequenced in this study. CTFV = Colorado tick fever virus.

**Table 3:** Serological evaluation of Colorado tick fever virus strains.

	Plaque Reduction Neutralization Test 90%			
Hyperimmune Fluid Antibody	Salmon River Virus ^1^	CTFV-Florio	California Hare Coltivirus	Eyach Virus ^3^
Salmon River	**640** ^2^	320	<10	10
CTFV Florio	640	**5120**	<10	10
California hare coltivirus	20	<10	**160**	<10

^1^ Bold text shows virus neutralization titers with homologous antibody. ^2^ Reciprocal endpoint neutralization titers. <10 is negative neutralization. ^3^ Homologous hyperimmune fluid was unavailable for Eyach virus. CTFV = Colorado tick fever virus.

## Data Availability

Sequences generated in this study have been deposited into GenBank (Table 1).

## Supplementary Materials

The following are available online at https://www.mdpi.com/article/10.3390/diseases9040092/s1, Figure S1: Phylogenetic inference of the genus *Coltivirus*, Table S1: Segment percent identities.
