# Supplementary material for: Genomic Evaluation of the Genus Coltivirus Indicates Genetic Diversity among Colorado Tick Fever Virus Strains and Demarcation of a New Species
Source: Diseases. 2021 Dec 17;9(4):92. doi: 10.3390/diseases9040092 (PMC8700517; doi:10.3390/diseases9040092)

Figure S1: Bayesian Maximum Clade Credibility trees of Colorado tick fever virus strains and select Coltiviruses. Segment open-reading frames were codon aligned with ClustalW in Mega v7. Bayesian phylogenies were inferred using BEAST v 1.8.4 with 50 million MCMC. Taxa are labeled with virus name, strain, segment number, and GenBank accession number. Branches are depicted with posterior probabilities, and scale bar indicates nucleotide substitutions per site.

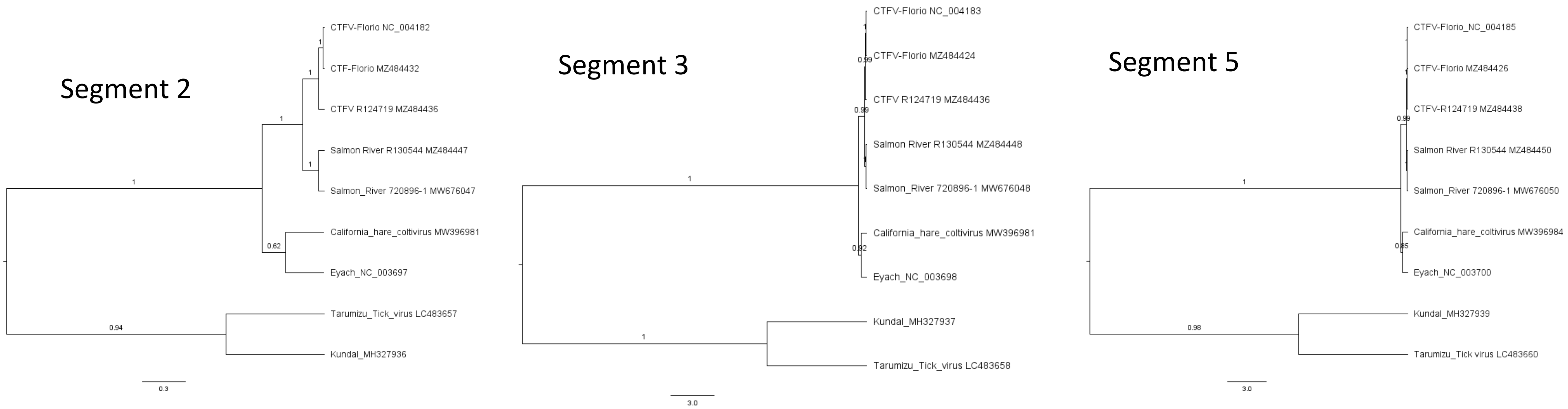

Segment 6

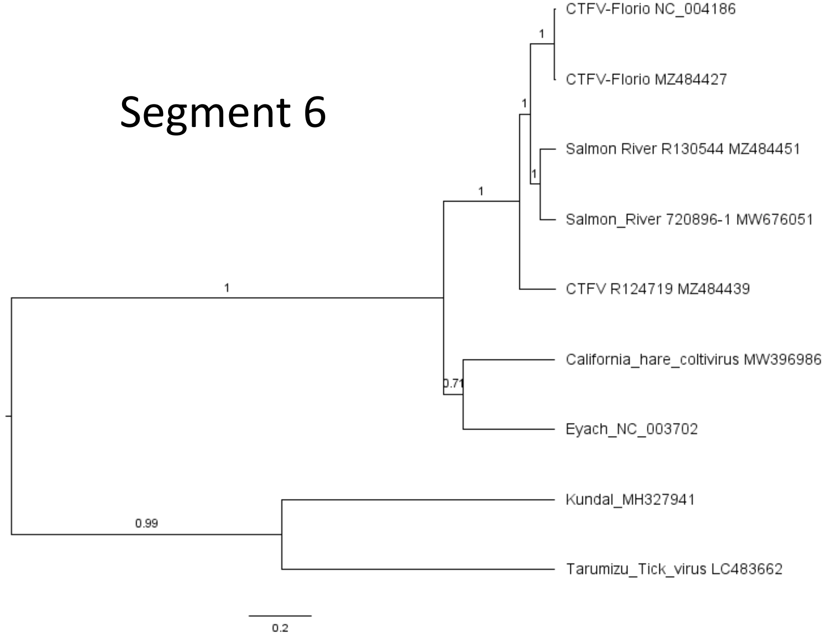

Segment 7

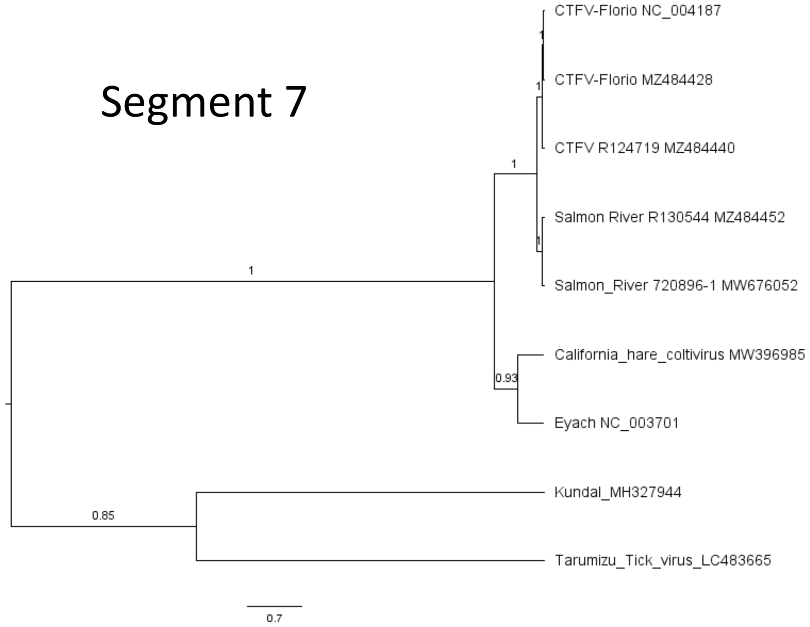

Segment 8

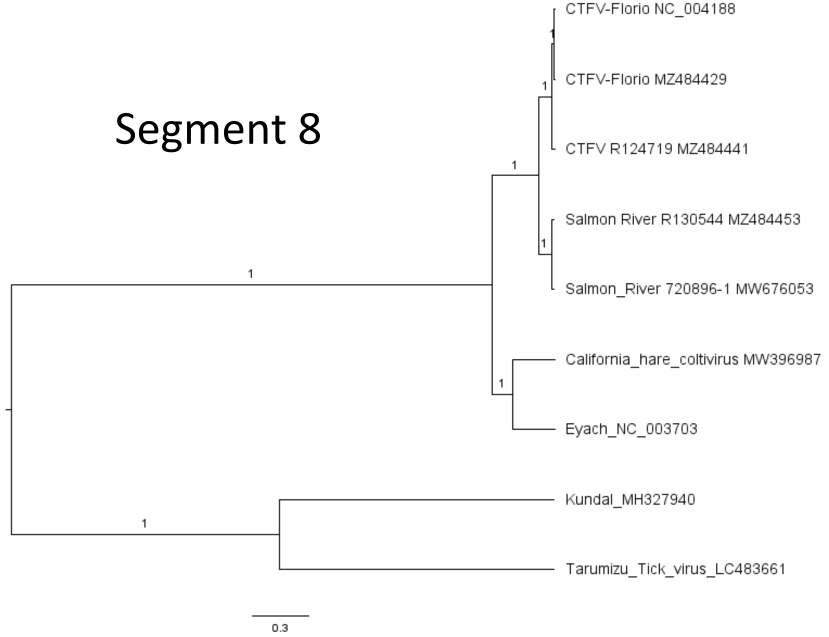

Segment 9

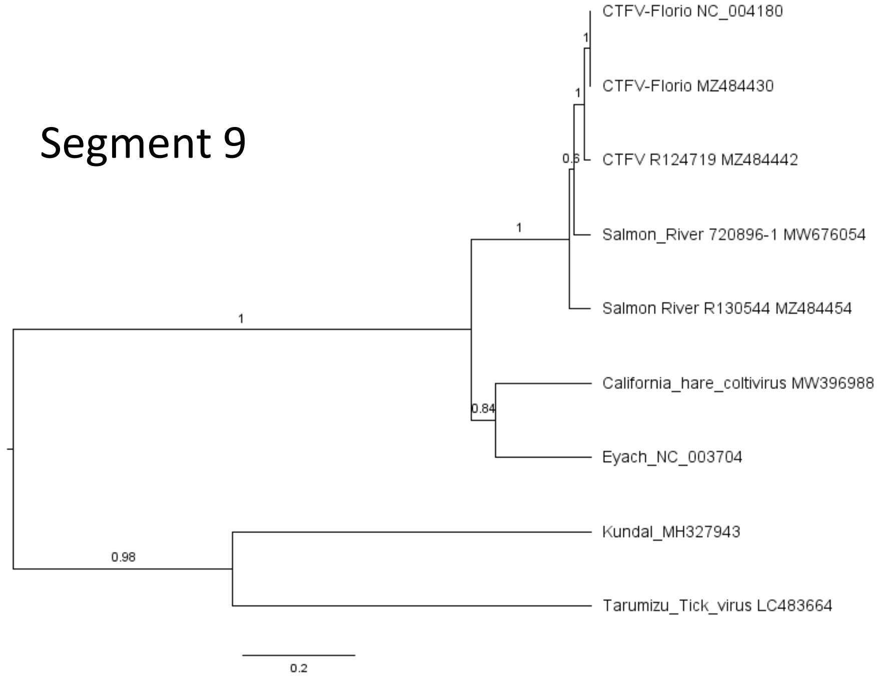

Segment 10

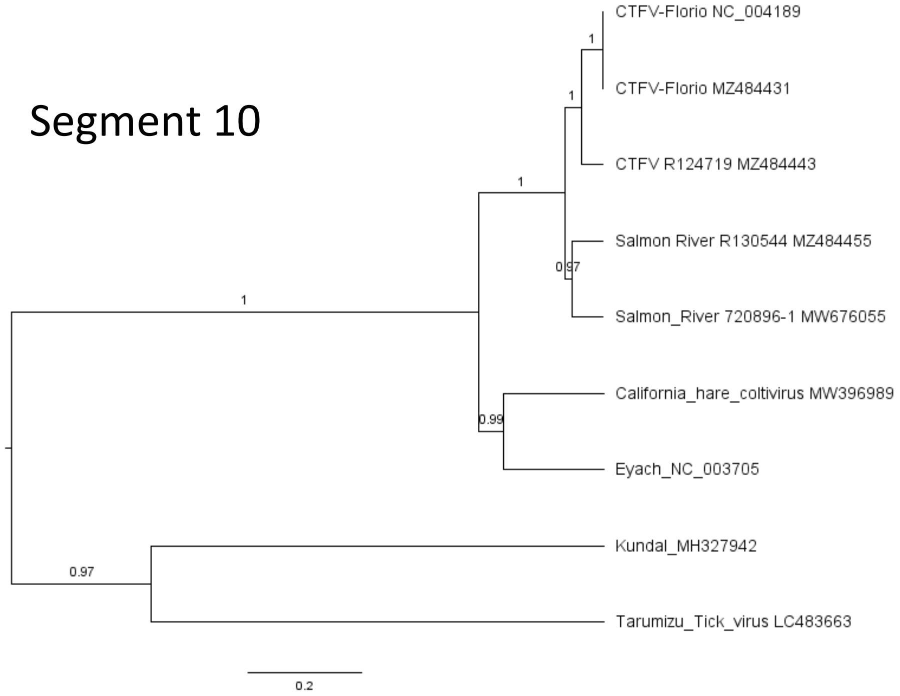

Segment 11

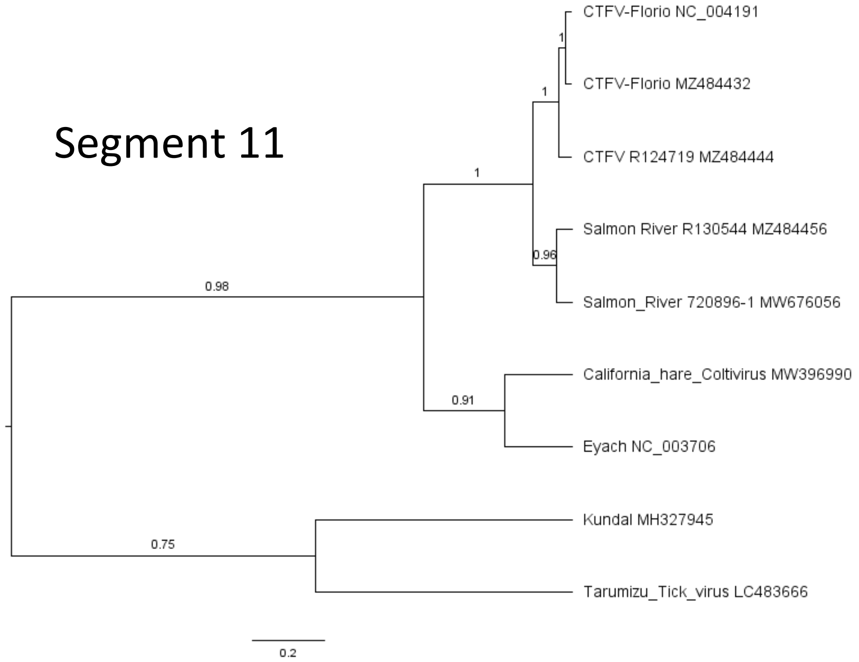

Segment 12

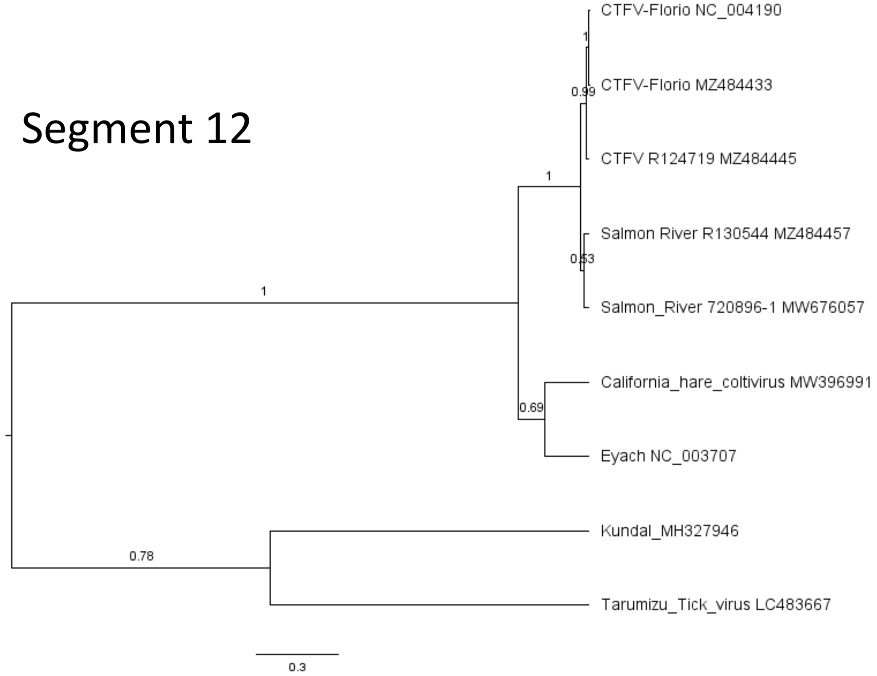

Supplement: Supplementary file 1 [file diseases-09-00092-s001.zip › Figure S1.pdf]
